# A Novel Intelligent Scan Assistant System for Early Pregnancy Diagnosis by Ultrasound: Clinical Decision Support System Evaluation Study

**DOI:** 10.2196/14286

**Published:** 2019-07-03

**Authors:** Ferdinand Dhombres, Paul Maurice, Lucie Guilbaud, Loriane Franchinard, Barbara Dias, Jean Charlet, Eléonore Blondiaux, Babak Khoshnood, Davor Jurkovic, Eric Jauniaux, Jean-Marie Jouannic

**Affiliations:** 1 Service de Médecine Fœtale Sorbonne Université Assistance Publique - Hôpitaux de Paris / Hôpitaux Universitaires Est Parisiens, Hôpital Armand Trousseau Paris France; 2 Medical Informatics and Knowledge Engineering for eHealth Lab INSERM Paris France; 3 Direction de la Recherche et de l'Innovation Assistance Publique - Hôpitaux de Paris Paris France; 4 Service de Radiologie Sorbonne Université Assistance Publique - Hôpitaux de Paris / Hôpitaux Universitaires Est Parisiens, Hôpital Armand Trousseau Paris France; 5 Obstetrical, Perinatal and Pediatric Epidemiology Research Team Center for Biostatistics and Epidemiology INSERM Paris France; 6 Gynaecology Diagnostic and Outpatient Treatment Unit University College Hospital and Institute for Women's Health University College London London United Kingdom

**Keywords:** decision support system, ontology, knowledge base, medical ultrasound, ectopic pregnancy

## Abstract

**Background:**

Early pregnancy ultrasound scans are usually performed by nonexpert examiners in obstetrics/gynecology (OB/GYN) emergency departments. Establishing the precise diagnosis of pregnancy location is key for appropriate management of early pregnancies, and experts are usually able to locate a pregnancy in the first scan. A decision-support system based on a semantic, expert-validated knowledge base may improve the diagnostic performance of nonexpert examiners for early pregnancy transvaginal ultrasound.

**Objective:**

This study aims to evaluate a novel Intelligent Scan Assistant System for early pregnancy ultrasound to diagnose the pregnancy location and determine the image quality.

**Methods:**

Two trainees performed virtual transvaginal ultrasound examinations of early pregnancy cases with and without the system. The ultrasound images and reports were blindly reviewed by two experts using scoring methods. A diagnosis of pregnancy location and ultrasound image quality were compared between scans performed with and without the system.

**Results:**

Each trainee performed a virtual vaginal examination for all 32 cases with and without use of the system. The analysis of the 128 resulting scans showed higher quality of the images (quality score: +23%; *P*<.001), less images per scan (4.6 vs 6.3 [without the CDSS]; *P*<.001), and higher confidence in reporting conclusions (trust score: +20%; *P*<.001) with use of the system. Further, use of the system cost an additional 8 minutes per scan. We observed a correct diagnosis of pregnancy location in 39 (61%) and 52 (81%) of 64 scans in the nonassisted mode and assisted mode, respectively. Additionally, an exact diagnosis (with precise ectopic location) was made in 30 (47%) and 49 (73%) of the 64 scans without and with use of the system, respectively. These differences in diagnostic performance (+20% for correct location diagnosis and +30% for exact diagnosis) were both statistically significant (*P*=.002 and *P*<.001, respectively).

**Conclusions:**

The Intelligent Scan Assistant System is based on an expert-validated knowledge base and demonstrates significant improvement in early pregnancy scanning, both in diagnostic performance (pregnancy location and precise diagnosis) and scan quality (selection of images, confidence, and image quality).

## Introduction

### Background

Ectopic pregnancy (EP) is defined by implantation of the gestational sac outside the endometrial cavity and occurs in 1%-2% of all pregnancies [1]. EP accounts for approximately 3%-5% of pregnancy-related deaths in developed countries [2]. Around 95% of EPs implant in the fallopian tubes and 5%-7% implant within the uterine wall but outside the uterine cavity. Nontubal EPs are more difficult to diagnose than tubal EP and are associated with a higher mortality and morbidity [3]. Delayed diagnosis is the main factor for EP associated with maternal death [4] and affects the success rate of future pregnancies [5]. Skilled ultrasound operators can diagnose an EP at an early stage by transvaginal sonography (TVS), often at the first examination [6]. However, less experienced operators perform first-line screening for patients at risk of EP in most emergency units; for them, this diagnosis remains difficult and more than three examinations are often needed [7].

### Prior Work

We have developed the first Intelligent Scan Assistant System for early pregnancy TVS examination. This clinical decision support system (CDSS) [8,9] is a computer program that provides diagnosis assistance during TVS examination of pregnancy of unknown location. During an ultrasound examination and in real time, this system assists the operator by suggesting ultrasound views to acquire and relevant signs to look for; it also displays reference ultrasound images demonstrating these relevant signs and views (from expert-reviewed collections of early pregnancy cases). The semantic design and features of this CDSS have been published in the medical semantics informatics community [10]. One key feature of this system is the personalized imaging protocol [11]: The system guides the operator through a structured acquisition of decisive ultrasound images to optimize the diagnostic pathway. We deemed this system “intelligent” because these personalized imaging protocols are not precalculated, but dynamically derived by the system (by SPARQL queries on the early pregnancy ontology of the knowledge base) from the guided image analysis of the current case.

In a preliminary study, this novel system demonstrated efficient support for a precise ultrasound image analysis, with a precision of 83% for the identification of signs in a series of 208 retrospectively collected ultrasound images of various types of ectopic pregnancies [10,11].

### Study Goal

In this study, we aimed to assess the added value of this novel CDSS for early pregnancy ultrasound. Our objective was to evaluate the effect of using the CDSS during TVS on scan quality and accuracy of the diagnosis of pregnancy location.

## Methods

### Clinical Decision Support System Evaluation Overview

Two obstetrics and gynecology (OB/GYN) trainees with basic national training in ultrasound imaging (including early pregnancy courses and simulation sessions) performed 32 ultrasound examinations in early pregnancy patients without and with the CDSS. These were re-examinations of prospectively collected 3D volumes from ultrasound data from the gynecology emergency unit at a university hospital. At the beginning of the study, the two trainees viewed a 2-minute video presentation of the CDSS and had a 10-minute hands-on session with the team who developed the CDSS. The TVS of early pregnancy cases was performed using a simulation device with and without the CDSS.

### Ultrasound (Transvaginal) Simulator and 3D Ultrasound Volume Collection

**Figure 1:**
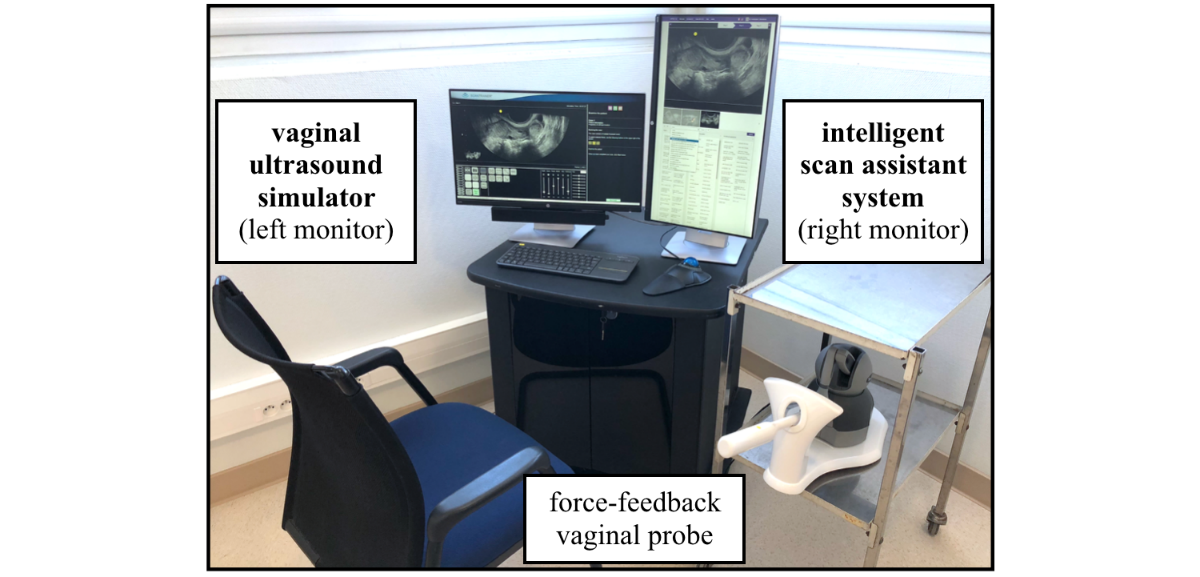
Global view of the virtual vaginal ultrasound platform for evaluation of the Intelligent Scan Assistant System. The left monitor displays the ultrasound simulator interface (ScanTrainer, MedaPhor, Wales, United Kingdom) and the right monitor displays the decision support system (Intelligent Scan Assistant System).

**Figure 2:**
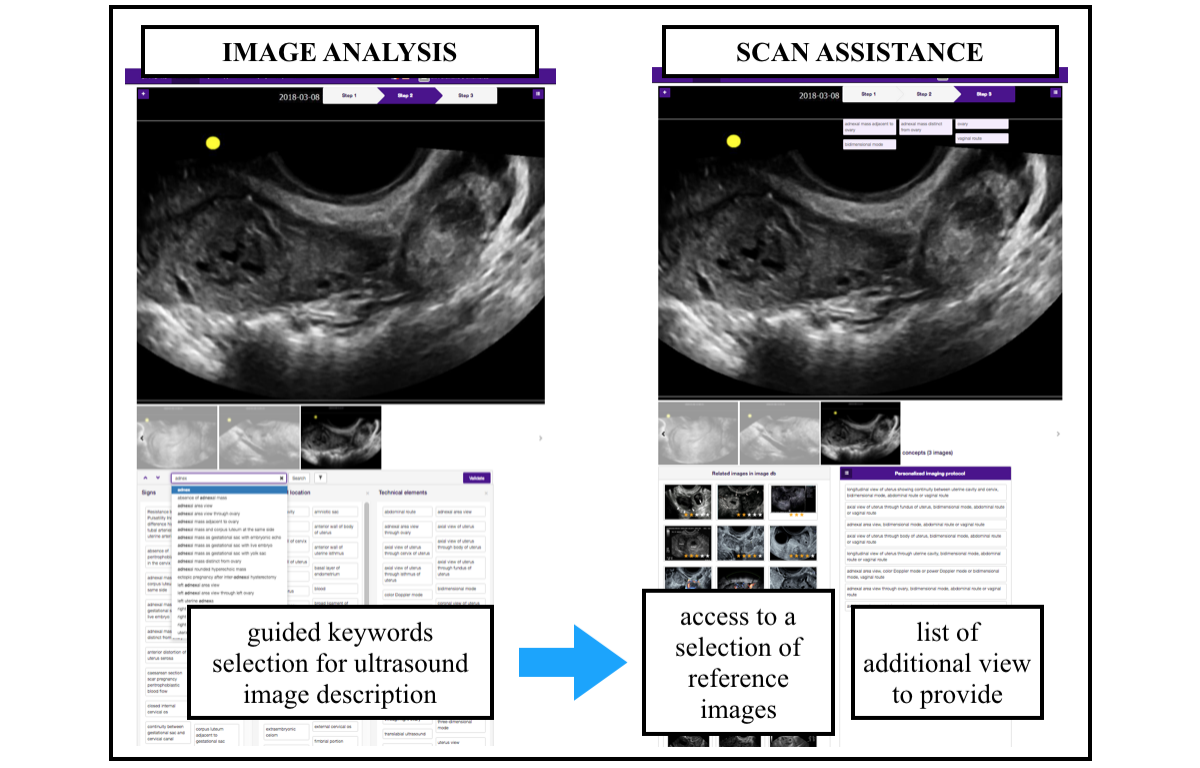
Detailed view of the Intelligent Scan Assistant System (right monitor). The two main steps with the decision support system on the right monitor are image analysis and scan assistance.

The simulation device was the interpolative model-based ultrasound simulator ScanTrainer (MedaPhor, Wales, United Kingdom) with a realistic haptic feedback transvaginal probe. This ultrasound simulator produces 2D images generated from 3D vaginal ultrasound volumes, which had been acquired during actual vaginal scans [12]. The complete virtual TVS platform used for the study integrates the ultrasound simulator and the CDSS with a dual screen setting (Figure 1). One screen displays the usual information for scanning as a regular ultrasound system. The other screen displays the CDSS interface for the image analysis and scan assistance (Figure 2).

**Figure 3:**
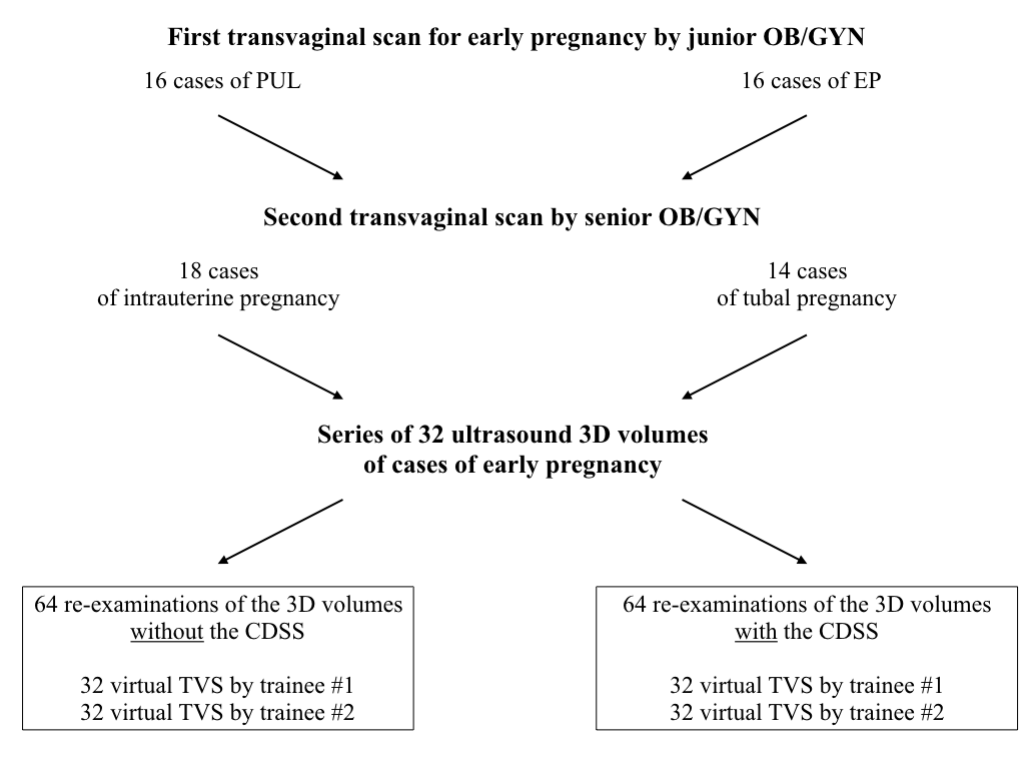
Three-dimensional ultrasound volume acquisition flowchart. Selection of cases for the 3D ultrasound volume series used for the virtual transvaginal scans (TVS) in this study. CDSS: clinical decision support system; EP: ectopic pregnancy; OB/GYN: obstetrics and gynecology; PUL: pregnancy of unknown locations.

Thirty-two 3D vaginal ultrasound volumes for this study were collected from patients during early pregnancy emergency examinations in one university hospital center (Figure 3), using an expert 3D ultrasound system (GE Healthcare Voluson E10/E8 with a RIC5-9-D vaginal probe, Cincinnati, OH). In our center, the first ultrasound examinations are always performed by junior OB/GYN examiners. In case of pregnancy of unknown locations or EP, a second vaginal scan is performed by a senior OB/GYN examiner. We collected 16 consecutive cases of pregnancy of unknown locations and 16 consecutive cases of EP diagnosed after the first ultrasound examination. For this study, three additional 3D volume acquisitions were performed by the senior OB/GYN examiner during the second TVS (acquisition field of 180°/100°): one volume for the uterus and one adjacent volume for each adnexal region. In case of a suspected ruptured EP, the volume acquisition was not performed, to avoid any delay in performing the surgical procedure. Additionally, when the diagnosis of intrauterine pregnancy was obvious (normal pregnancy of 6 weeks of gestation or more) at the second examination by the senior, no 3D volume was acquired. Rare types of ectopic pregnancy (heterotopic pregnancy, interstitial pregnancy, caesarean-section scar pregnancy, and cervical pregnancy) were also excluded from this study. Thus, a consecutive series of 32 sets of 3D vaginal ultrasound volumes was collected, deidentified, and imported in the ultrasound simulator. The final diagnoses of the 32 cases in this series were intrauterine pregnancy (n=18) and tubal EP (n=14), all correctly diagnosed by senior TVS experts and confirmed by pregnancy outcomes.

### Clinical Decision Support System Evaluation Protocol

#### Virtual Ultrasound Examinations

**Figure 4:**
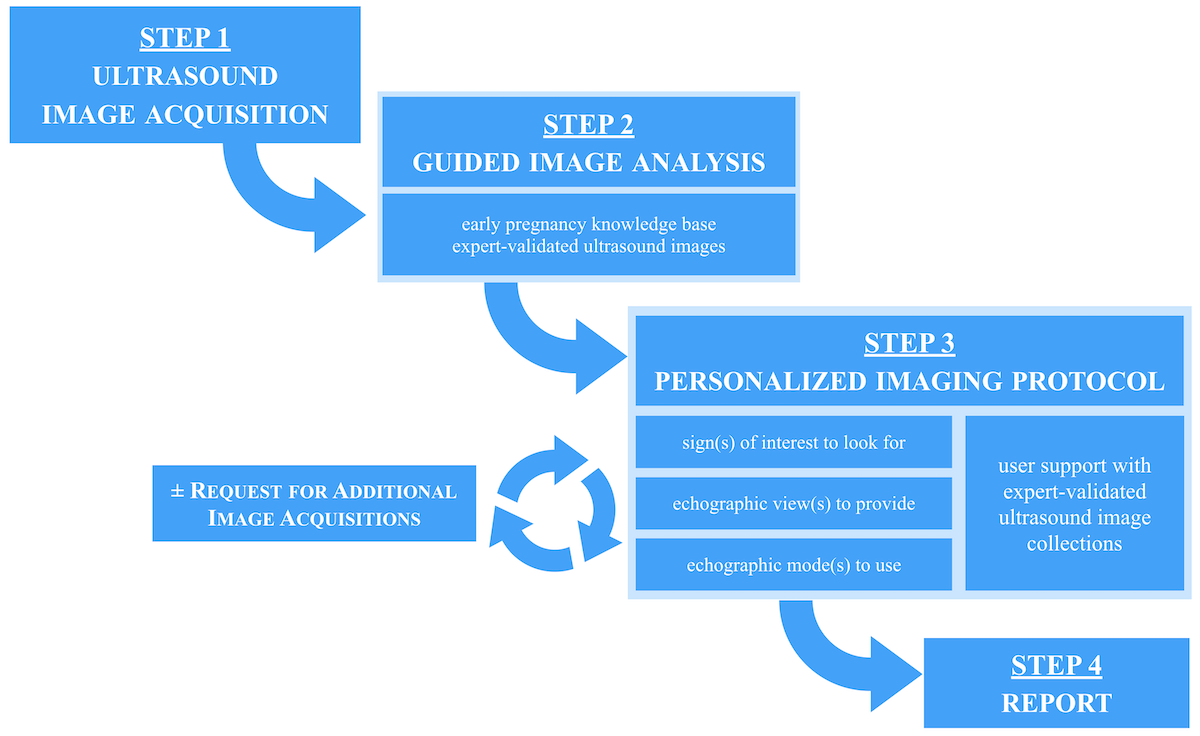
Personalized imaging protocol and workflow of the Intelligent Scan Assistant System for ultrasound imaging.

The two trainees performing the virtual TVS were independent of acquisition of 3D volumes, and they were unaware of the medical report and final diagnosis. The clinical information provided for the scans were identical for all cases and limited to “moderate pelvic pain and positive pregnancy test.” The 32 scans were performed twice by each trainee without supervision in a random order in a nonassisted mode (without the CDSS) and 2 months later in assisted mode (with the CDSS). The potential recall bias was also controlled by the 2-month interval between the two TVS sessions. Additionally, it should be mentioned that during these 2 months, the two trainee operators did not receive any ultrasound training and did not have any ultrasound scanning activity. In the nonassisted mode, the scans were performed following the usual protocol for OB/GYN emergency ultrasound in our center [13,14], using a standardized reporting system. In the assisted mode, the scans were performed following a personalized image analysis and acquisition protocol suggested by the CDSS [10,11]. The personalized imaging protocol and the CDSS workflow are presented in Figure 4. Briefly, in step 1, the operator performs the scan and acquires ultrasound images. In step 2, he/she follows the system guidance for a precise analysis of these acquired images: He/she describes the image with keywords for anatomical structures, ultrasound signs, and technical elements (ultrasound route, mode, and view). The keywords are displayed with text definitions and are illustrated by expert-validated images. In step 3, if necessary, the system suggests providing additional imaging elements (ultrasound views, signs, and anatomical structures), thus assisting the operator in establishing a comprehensive image set in order to reach a precise diagnosis. This is a personal imaging protocol that is automatically calculated by the system. The personal imaging protocol is derived from computer-based reasoning over the early pregnancy knowledge base. After step 3, the user may either follow the personal imaging protocol and provide the additional requested elements or proceed to the final report and finish the examination (step 4).

#### Ultrasound Images and Report Scoring Methods

For each examination, we collected the images, reports, and data on duration of the scans. Two senior experienced ultrasound operators reviewed the images and reports of the virtual examinations. During the review, they were blinded to the use of the CDSS and the final diagnosis. They had the same minimal clinical information for all cases as the two trainees: “moderate pelvic pain and positive pregnancy test.”

They scored the images according to the quality criteria for the sagittal view of the uterus and the ovaries as per a previous study [14]. The maximum quality score was 15 points (Textbox 1). They also performed a subjective scoring of the scans and reports, reflecting their trust in the conclusion of the report associated with the images. This level of trust was assessed with a 5-level scale (Textbox 2).

**The image set quality was assessed using a score based on 15 items.**

**On the sagittal view of the uterus, the five quality criteria were as follows:**

- “uterine cervix visible” (1 point)
- “uterine fundus visible” (1 point)
- “endometrial midline echo visible” (1 point)
- “endocervix visible” (1 point)
- “uterus occupying more than half of the total image size” (1 point)

**On each of the views of the ovaries, the 5×2 quality criteria were as follows:**

- “side stated” (1 point)
- “follicle(s) visible” (1 point)
- “iliac vein visible” (1 point)
- “long axis of the ovary <30° with the horizontal line” (1 point)
- “vary occupying more than a quarter of the total image size” (1 point)

**The level of trust in the report was assessed using a 5-level scale.**

- **Level 1: No trust in the final diagnosis (incorrect):** The diagnosis is most likely incorrect, and the image set suggests another diagnosis. Immediate supervisor examination is needed.
- **Level 2: No trust in the final diagnosis (low quality):** The image set quality is insufficient and/or does not support the final diagnosis. Immediate supervisor examination is needed.
- **Level 3: Moderate trust in the final diagnosis:** Although the diagnosis might be correct, the image set quality is insufficient, and a supervisor examination is needed.
- **Level 4: The image set quality could be improved**; however, it is of sufficient quality to accurately support the final diagnosis. No supervisor examination is needed.
- **Level 5 represents a total trust** **in the final diagnosis**: The image set effectively supports the diagnosis, and no supervisor examination is needed.

### Statistical Analysis

The reproducibility of the scoring methods for quality and trust was assessed on a random sample of 20% of all scans (n=25) and independently reviewed by both experts. We tested for the differences in trust and quality scores. We also tested for the differences between examination modes (assisted vs nonassisted mode) in the diagnosis of location of pregnancy (ectopic OR nonectopic) and in the final diagnosis precision (exact location of the ectopic pregnancy, ie, “tubal pregnancy” explicitly stated in the report conclusion). The gold standard for the diagnosis was the final diagnosis in all cases, as reported in the senior TVS report and confirmed by the pregnancy follow-up.

Statistical analysis was performed using R, version 3.3.1 (R Foundation for Statistical Computing, Vienna, Austria) and STATA, version 15 (StataCorp, College Station, TX). Paired *t* tests were performed to compute the difference in continuous variables (scan duration, image count, quality score, and trust score). Exact McNemar tests were used to test for the differences in categorical variables (presence of the three mandatory ultrasound views, diagnosis of location, and final diagnosis precision). We also calculated differences in the proportions of outcomes, with 95% CIs, for assisted versus nonassisted modes. Adjusted kappa coefficients (Cohen weighted kappa) for quality scores and trust scores were computed to test for the reproducibility of the scoring methods.

For all tests, a *P* value ≤.05 was considered statistically significant. Adjusted kappa values <0.6, between 0.6 and 0.8, and >0.8 were considered to represent poor, moderate, and good agreement, respectively.

### Ethics Approval

The development of this CDSS for early pregnancy (including expert-validated early pregnancy ultrasound images) and the evaluation study (including collection and analysis of 3D ultrasound volumes of early pregnancy) were both approved by the French National College of the OB/GYN Institutional Review Board (CNGOF Research Ethics Committee CEROG #2015-GYN-1002 and #2016-GYN-0601, respectively).

## Results

### Virtual Scans and Scoring Method Reproducibility

Each trainee performed a virtual transvaginal examination for all 32 cases with and without the system. The expert operators reviewed the 128 resulting scans for quality of images and trust in the reports. The experts’ agreement was tested on 25 scans. The level of agreement was good, with kappa values of 0.86 (0.76-0.96) for objective quality scoring and 0.86 (0.70-1.0) for subjective trust scoring.

### Impact of the Clinical Decision Support System on Image Quality

The scan quality differences are presented in Table 1. We found that the average quality score for ultrasound images was 23% higher when using the CDSS than with the nonassisted mode, with an average value of 12.6 of 15 (*P*<.001). Additionally, when using the CDSS, the number of images per scan was lower than that with the nonassisted mode (4.6 vs 6.3, *P*<.001).

The average trust score was 20% higher when using the CDSS than with the nonassisted mode, with an average value of 4.12 of 5 (*P*<.001).

**Table 1:** Differences in scan quality with (assisted mode) and without (nonassisted mode) the decision support system.^a^

Scan quality parameter	Assisted mode (64 scans)	Nonassisted mode (64 scans)	Difference	*P* value
Image count in report, mean (SD)	4.64 (0.80)	6.33 (2.07)	–1.69 (–27%)	<.001
Scan duration (minutes), mean (SD)	14.7 (7.1)	6.4 (3.3)	+8.3 (+129%)	<.001
Quality score of image sets, mean (SD)	12.5 (1.86)	10.2 (1.90)	+2.3 (+23%)	<.001
Trust score of report, mean (SD)	4.12 (0.83)	3.42 (1.04)	+0.70 (+20%)	<.001

^a^The tests for difference were paired *t* tests.

### Impact of the Clinical Decision Support System on Diagnosis of Pregnancy Location

The diagnosis differences are displayed in Table 2. We observed a correct diagnosis of location in 39 (61%) and 52 (81%) of 64 scans in the nonassisted mode and assisted mode, respectively. Additionally, the exact diagnosis was achieved in 30 (47%) and 49 (77%) scans in the nonassisted mode and assisted mode, respectively. These differences (+20% for correct location diagnosis and +30% for exact diagnosis) were both statistically significant (*P*=.002 and *P*<.001, respectively).

Without the use of the CDSS, we recorded 8 false-negative diagnoses of tubal EP (cases 44 and 23 for both trainees and cases 50, 45, 33, and 1 for one trainee). With the CDSS, the false-negative result for ectopic pregnancy was a scan of a tubal pregnancy case (case 44 for one trainee). In the other seven cases with false-negative diagnoses of EP, all relevant signs associated with the final diagnosis (with reference images) were presented to the trainee when using the CDSS. More precisely, images exhibiting the key features of tubal pregnancy were acquired following the personalized protocol of the CDSS and correctly diagnosed. Additionally, the quality score, trust score, and number of images per scan were significantly different when using the CDSS as compared to not using the CDSS: 12.6 versus 10.2 (*P*=.01), 4.0 versus 3.0 (*P*=.02), and 4.7 versus 6.5 (*P*=.04), respectively. The scan duration was also significantly different when using the CDSS as compared to not using the CDSS (14.4 versus 7.6 min; *P*=.003).

**Table 2:** Differences in the diagnostic performance of trainees with (assisted mode) and without the decision support system (nonassisted mode).^a^

Diagnostic performance parameter	Assisted mode (64 scans), n (%)	Nonassisted mode (64 scans), n (%)	Difference, n	Difference, % (95% CI)	*P* value
Correct pregnancy location (ectopic/nonectopic)	52 (81)	39 (61)	13	+20 (7-33)	.002
Exact diagnosis (with precise ectopic location)	49 (77)	30 (47)	19	+30 (15-44)	<.001
False-negative of ectopic pregnancy	1 (1.6)	8 (12.5)	–7	–10.9	—^b^
False-positive of ectopic pregnancy	3 (4.7)	3 (4.7)	0	—	—

^a^The test for difference was exact McNemar test.

^b^Not available.

We observed 3 false-positive EP diagnoses in the assisted mode and 3 other false-positive EP diagnoses in the nonassisted mode, which were the 6 cases of intrauterine pregnancies.

## Discussion

### Principal Results

Our study demonstrated a significant improvement in early pregnancy ultrasound examination, both in diagnostic performance (pregnancy location and precise diagnosis) and scan quality (selection of images, confidence, and image quality) for OB/GYN trainees using the CDSS, when pregnancy of unknown locations or EP was suspected.

Definitive diagnosis of ectopic pregnancy can be achieved by TVS, but it relies on a precise analysis of ultrasound findings [1,15-19]. However, in most emergency units, the initial TVS is usually performed by trainees or sonographers with basic expertise in OB/GYN scanning. The support of expert-validated images in addition to the personalized protocol (intelligent suggestions of ultrasound signs, views, and modes) are key features of the CDSS, especially for improving the false-negative diagnoses of EP. During the examination, it provides actionable knowledge to less experienced operators, thus improving their diagnostic capabilities in real time. Our results suggest that the CDSS improves not only the diagnosis of early pregnancy location, but also the diagnostic accuracy. The use of the CDSS resulted in a better selection of images with higher quality. This facilitates the review of the scans by the senior experts in our department, as suggested by their higher trust scores.

### Limitations

The main limitation of our study is that our evaluation relies on virtual TVS. In a previous study, Infantes et al [20] showed that offline analysis of 3D TVS static datasets has limitations in terms of the diagnostic accuracy for EP [20]. However, their study was not conducted with ultrasound simulation platforms [12,21]. In our study, we chose the best simulation options for realistic 2D ultrasound examinations. This led to a moderate loss in image quality, but with this study design, the same patient would have been scanned twice (with and without the CDSS) and by each trainee (32 patients, 128 scans; 4 scans per patient), thus specifically assessing the potential added value of the CDSS itself. A key skill in ultrasound is to find the pathology and, in the simulator, the trainees were presented with volumes that contained all necessary information to make a diagnosis; therefore, their scanning skills were not properly evaluated in this study. However, even if the interpretation was easier, we believe this was not a bias in favor of the CDSS. Interestingly, the trainees complained about the lack of color Doppler imaging (CDI) in the simulator, but only when they used the CDSS and, in particular, for the cases of false-positive diagnoses of EP. Better ultrasound imaging quality and access to the CDI mode might change the performances of the CDSS. As the CDSS includes a rich CDI semiology of EP, this change might even be in favor of the CDSS. Palpation by a transvaginal probe often provides critical information to make the correct diagnosis. This cannot be done on current simulators, which is another limitation of the study.

We observed an increase of 8 minutes in the scan duration. Similar additional time costs were observed in a pilot study when using standardized protocols with integrated software for the second trimester screening [22]. Consequently, we believe that technical improvements, in particular, integration in the ultrasound platform, could improve examination durations; in our study, each image file was manually imported in the CDSS during the TVS. Overall, an additional 8 minutes is a reasonable cost for significant diagnostic improvements, and consequently, for a reduction in the number of visits to diagnose the correct location of the pregnancy.

### Comparison With Prior Work

This CDSS is the first computer-based reasoning system in the field of OB/GYN. In the current trend of new technical solutions to improve ultrasound examinations, this CDSS has been evaluated, offers novel intelligent scan assistance in real time, is dynamically based on previous ultrasound findings, and has salient reference images in backup. In contrast, only 58% of CDSSs demonstrated improvements in the processes of care [23].

Software-enforced standardized protocols for screening offer interesting solutions for ultrasound scan improvement [22]. These systems implement static checklists to improve the acquisition of standard image sets. Other tools automated the 2D images processing (eg, for caliper positioning) and the 3D/4D volume processing (eg, to derive 2D images of the fetal brain and heart) [24-26]. Finally, online resources provide access to collections of medical images, including ultrasound and OB/GYN material [27-29].

**Figure 5:**
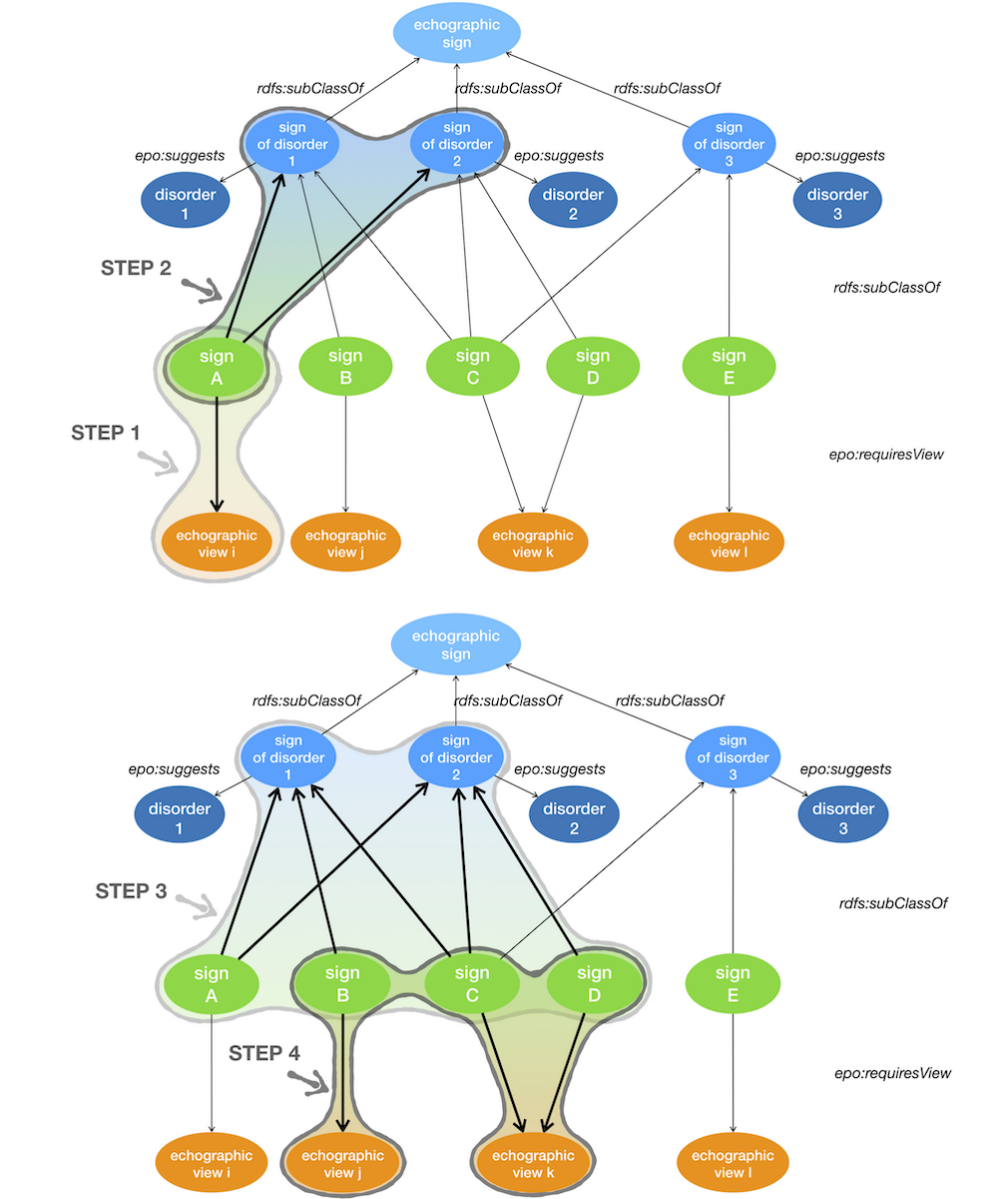
Principles of clinical reasoning for the CDSS represented in the ontology for early pregnancy (epo). Step 1: Identification of "epo:sign_A", using "epo:echographic_view_i". Step 2: Compute the list of disorders suggested by "epo:sign_A," "epo:disorder_1," and "epo:disorder_2". Step 3: Compute the list of signs for the list of disorders: "epo:sign_B," "epo:sign_C," and "epo:sign_D". Step 4: Suggest a list of echographic views required for the list of signs: "epo:echographic_view_j" and "epo:echographic_view_k". CDSS: clinical decision support system.

Our choice of a CDSS based on ontology and semantic Web technologies have several significant advantages. The ontology is a model representing the medical knowledge involved in TVS for early pregnancy. This model allows computer-based reasoning and enables the personalized imaging protocol feature of the CDSS. Of particular interest, this type of ontology-based reasoning CDSS differs from current systems (eg, deep learning and neural network systems) and does not integrate any “black-box” component [9]: Every step of the calculation can be audited and is readable by a human. More specifically, Figure 5 illustrates the effective support of the ontology to derive a personalized imaging protocol. Every step of the protocol relies on SPARQL queries to navigate through the graph of the knowledge base (XML/RDF). This knowledge base is present in a triplestore with semantic inference capabilities (OWL/HermIT). Consequently, the result of every step of the protocol is a set of resource description framework triple, with labels (skos:prefLabel) that can be reviewed by medical experts.

When a sign is identified during the scan, in an echographic view, the clinical reasoning principle is formalized as follows:

1. List all disorders suggested by the identified sign(s).
2. For all these disorders, list all associated sign(s).
3. For all these signs, list all required echographic view(s).
4. Provide support to the operator: ordered list of echographic views required to look for relevant signs for differential diagnosis.

The CDSS design implements international standards (RDF, SPARQL, and OWL) with a generic strategy for medical imaging. The CDSS was initially developed for early pregnancy ultrasound. There is no technical obstacle to extending the system to other areas of ultrasound imaging such as diagnosis of placentation disorders or morphological ultrasound examination of the fetus. Furthermore, it allows for a simple curation process (eg, addition of new signs or new cases in the collection) and does not require specific skills in informatics. For example, when new ultrasound imaging features are described in medical publications (eg, when fetal ultrasound features of postnatal disorders are discovered, as it was recently the case for the limited dorsal myeloschisis, which is a well-known postnatal disorder [30]), updating the whole system is easy. In contrast, updating usual expert systems would require technical developments. Finally, semantic Web technologies are designed to scale and support interoperability. The scaling capability is the capability to handle a large amount of data and is a prerequisite to cover the large domain of fetal abnormalities, including ultrasound features, anatomical locations, adequate ultrasound views, and nosology of fetal disorders. The interoperability capability opens data integration with other databases, in particular, genetic data repositories. This interoperability is a possible way to establish correlations between ultrasound phenotypes and genetic variants [31].

### Conclusions

In the growing ecosystem of emerging new tools for medical imaging assistance, the Intelligent Scan Assistant System is a CDSS based on a semantic representation of expert knowledge, consistent with a complementary solution that promotes improvement of both scan quality and diagnostic accuracy. The evaluation of the system on a simulation platform demonstrated its added value for trainees in TVS. Consequently, the implementation of this CDSS in routine care may reduce the number of TVS examinations to the minimum number of TVSs required to diagnose (or exclude) EP. These results await confirmation by randomized control trials and further use in different areas of OB/GYN imaging.

## Abbreviations

CDI: color Doppler imaging
CDSS: clinical decision support system
EP: ectopic pregnancy
OB/GYN: obstetrics and gynecology
TVS: transvaginal sonography

## References

[1] Memtsa M, Jamil A, Sebire N, Jauniaux E, Jurkovic D (2013). Diagnosis and management of intramural ectopic pregnancy. Ultrasound Obstet Gynecol.

[2] Creanga AA, Syverson C, Seed K, Callaghan WM (2017). Pregnancy-Related Mortality in the United States, 2011-2013. Obstet Gynecol.

[3] Knez J, Day A, Jurkovic D (2014). Ultrasound imaging in the management of bleeding and pain in early pregnancy. Best Pract Res Clin Obstet Gynaecol.

[4] Cantwell R, Clutton-Brock T, Cooper G, Dawson A, Drife J, Garrod D, Harper A, Hulbert D, Lucas S, McClure J, Millward-Sadler H, Neilson J, Nelson-Piercy C, Norman J, O'Herlihy C, Oates M, Shakespeare J, de Swiet M, Williamson C, Beale V, Knight M, Lennox C, Miller A, Parmar D, Rogers J, Springett A (2011). Saving Mothers' Lives: Reviewing maternal deaths to make motherhood safer: 2006-2008. The Eighth Report of the Confidential Enquiries into Maternal Deaths in the United Kingdom. BJOG.

[5] Ghaneie A, Grajo JR, Derr C, Kumm TR (2015). Unusual ectopic pregnancies: sonographic findings and implications for management. J Ultrasound Med.

[6] Jurkovic D, Wilkinson H (2011). Diagnosis and management of ectopic pregnancy. BMJ.

[7] Wedderburn CJ, Warner P, Graham B, Duncan WC, Critchley HOD, Horne AW (2010). Economic evaluation of diagnosing and excluding ectopic pregnancy. Hum Reprod.

[8] Shortliffe EH (1987). Computer programs to support clinical decision making. JAMA.

[9] Shortliffe EH, Sepúlveda MJ (2018). Clinical Decision Support in the Era of Artificial Intelligence. JAMA.

[10] Dhombres F, Maurice P, Friszer S, Guilbaud L, Lelong N, Khoshnood B, Charlet J, Perrot N, Jauniaux E, Jurkovic D, Jouannic JM (2017). Developing a knowledge base to support the annotation of ultrasound images of ectopic pregnancy. J Biomed Semantics.

[11] Maurice P, Dhombres F, Blondiaux E, Friszer S, Guilbaud L, Lelong N, Khoshnood B, Charlet J, Perrot N, Jauniaux E, Jurkovic D, Jouannic JM (2017). Towards ontology-based decision support systems for complex ultrasound diagnosis in obstetrics and gynecology. J Gynecol Obstet Hum Reprod.

[12] Chalouhi GE, Bernardi V, Ville Y (2015). Ultrasound simulators in obstetrics and gynecology: state of the art. Ultrasound Obstet Gynecol.

[13] Popowski T, Huchon C, Fathallah K, Bouhanna P, Bernard J, Fauconnier A (2012). Standardization of the gynecological emergency ultrasound examination. Gynecol Obstet Fertil.

[14] Salomon LJ, Nassar M, Bernard JP, Ville Y, Fauconnier A, Société Française pour l'Amélioration des Pratiques Echographiques (SFAPE) (2009). A score-based method to improve the quality of emergency gynaecological ultrasound examination. Eur J Obstet Gynecol Reprod Biol.

[15] Jurkovic D, Mavrelos D (2007). Catch me if you scan: ultrasound diagnosis of ectopic pregnancy. Ultrasound Obstet Gynecol.

[16] Barnhart K, van Mello NM, Bourne T, Kirk E, Van Calster B, Bottomley C, Chung K, Condous G, Goldstein S, Hajenius PJ, Mol BW, Molinaro T, O'Flynn O'Brien KL, Husicka R, Sammel M, Timmerman D (2011). Pregnancy of unknown location: a consensus statement of nomenclature, definitions, and outcome. Fertil Steril.

[17] Kirk E, Bottomley C, Bourne T (2014). Diagnosing ectopic pregnancy and current concepts in the management of pregnancy of unknown location. Hum Reprod Update.

[18] [No author listed] (2018). ACOG Practice Bulletin No. 193: Tubal Ectopic Pregnancy. Obstet Gynecol.

[19] Nadim B, Infante F, Lu C, Sathasivam N, Condous G (2018). Morphological ultrasound types known as 'blob' and 'bagel' signs should be reclassified from suggesting probable to indicating definite tubal ectopic pregnancy. Ultrasound Obstet Gynecol.

[20] Infante F, Espada Vaquero M, Bignardi T, Lu C, Testa AC, Fauchon D, Epstein E, Leone FPG, Van den Bosch T, Martins WP, Condous G (2018). Prediction of Tubal Ectopic Pregnancy Using Offline Analysis of 3-Dimensional Transvaginal Ultrasonographic Data Sets: An Interobserver and Diagnostic Accuracy Study. J Ultrasound Med.

[21] Chalouhi GE, Bernardi V, Gueneuc A, Houssin I, Stirnemann JJ, Ville Y (2016). Evaluation of trainees' ability to perform obstetrical ultrasound using simulation: challenges and opportunities. Am J Obstet Gynecol.

[22] Bultez T, Bernard J, Metzger U, Ville Y, Salomon LJ (2017). Pilot Study of a Software-Supported Protocol for Second-Trimester Ultrasound Screening. J Ultrasound Med.

[23] Roshanov PS, Fernandes N, Wilczynski JM, Hemens BJ, You JJ, Handler SM, Nieuwlaat R, Souza NM, Beyene J, Van Spall HG, Garg AX, Haynes RB (2013). Features of effective computerised clinical decision support systems: meta-regression of 162 randomised trials. BMJ.

[24] Yeo L, Romero R (2016). Intelligent navigation to improve obstetrical sonography. Ultrasound Obstet Gynecol.

[25] Garcia M, Yeo L, Romero R, Haggerty D, Giardina I, Hassan SS, Chaiworapongsa T, Hernandez-Andrade E (2016). Prospective evaluation of the fetal heart using Fetal Intelligent Navigation Echocardiography (FINE). Ultrasound Obstet Gynecol.

[26] Moratalla J, Pintoffl K, Minekawa R, Lachmann R, Wright D, Nicolaides KH (2010). Semi-automated system for measurement of nuchal translucency thickness. Ultrasound Obstet Gynecol.

[27] Tutschek B, Pilu G (2017). Pocket Brain, an interactive, web-based ultrasound atlas of normal and abnormal fetal brain development. Ultrasound Obstet Gynecol.

[28] Demner-Fushman D, Kohli MD, Rosenman MB, Shooshan SE, Rodriguez L, Antani S, Thoma GR, McDonald CJ (2016). Preparing a collection of radiology examinations for distribution and retrieval. J Am Med Inform Assoc.

[29] Kahn CE, Thao C (2007). GoldMiner: a radiology image search engine. AJR Am J Roentgenol.

[30] Friszer S, Dhombres F, Morel B, Zerah M, Jouannic JM, Garel C (2017). Limited Dorsal Myeloschisis: A Diagnostic Pitfall in the Prenatal Ultrasound of Fetal Dysraphism. Fetal Diagn Ther.

[31] Köhler S, Vasilevsky NA, Engelstad M, Foster E, McMurry J, Aymé S, Baynam G, Bello SM, Boerkoel CF, Boycott KM, Brudno M, Buske OJ, Chinnery PF, Cipriani V, Connell LE, Dawkins HJS, DeMare LE, Devereau AD, de Vries BBA, Firth HV, Freson K, Greene D, Hamosh A, Helbig I, Hum C, Jähn JA, James R, Krause R, F Laulederkind SJ, Lochmüller H, Lyon GJ, Ogishima S, Olry A, Ouwehand WH, Pontikos N, Rath A, Schaefer F, Scott RH, Segal M, Sergouniotis PI, Sever R, Smith CL, Straub V, Thompson R, Turner C, Turro E, Veltman MWM, Vulliamy T, Yu J, von Ziegenweidt J, Zankl A, Züchner S, Zemojtel T, Jacobsen JOB, Groza T, Smedley D, Mungall CJ, Haendel M, Robinson PN (2017). The Human Phenotype Ontology in 2017. Nucleic Acids Res.

